# Intensive Interaction for children and young people with profound and multiple learning disabilities (INTERACT): study protocol for a cluster randomised controlled trial, economic evaluation and process evaluation

**DOI:** 10.1186/s13063-026-09669-5

**Published:** 2026-04-10

**Authors:** Kerry J. Bell, Jill Bradshaw, Judy Clegg, Katie Whiteside, Kalpita Baird, Charlie Peck, Arabella Scantlebury, Han-I. Wang, Joy Adamson, Amanda Allard, Jane E. Blackwell, Katie Carlisle, Imogen Fountain, Nick Gore, Fliss Kyffin, Jennifer Miller, Adenike Okanlawon, Lindsay Pennington, Lyn Robinson-Smith, Sarah Ronaldson, Emma Standley, Joanne Sweeney, Lucy Ziegler, Catherine Hewitt

**Affiliations:** 1 York Trials Unit, Department of Health Sciences, University of York, York, UK; 2 Intellectual Disabilities Research Institute, University of Birmingham, Birmingham, UK; 3 School of Allied Health, Pharmacy, Nursing and Midwifery, University of Sheffield, Sheffield, UK; 4 Centre for Evidence and Implementation Science, University of Birmingham, Birmingham, UK; 5 Council for Disabled Children, London, UK; 6 School of Education, Bangor University, Bangor, UK; 7 Promoting a More Inclusive Society (PAMIS), Dundee, UK; 8 Population Health Sciences Institute, Newcastle University, Newcastle Upon Tyne, UK; 9 Centre for Health Economics, University of York, York, UK; 10 Patient and Public Involvement and Engagement Representative, Leeds, London UK

**Keywords:** Intensive interaction, Profound and multiple learning disabilities, Speech and language therapy, Communication, Education, Parent carers

## Abstract

**Background:**

Communication interventions can facilitate communication between people with profound and multiple learning disabilities (PMLD) and familiar partners such as family and educational setting staff, including speech and language therapists. Various communication interventions are routinely used but their clinical and cost-effectiveness are unclear. Intensive Interaction (II) is one intervention that focuses on early interaction abilities. II can be delivered by staff in educational settings and/or at home. Despite many settings already implementing II, staff are sometimes untrained or have not received up to date training, potentially leading to inconsistencies in how the technique is applied and the quality of the interactions. We will provide structured training in II to educational setting staff and parents/carers with coordinated activities developed jointly for each child/young person to be delivered within the educational setting and at home. This study aims to establish whether Intensive Interaction delivered within educational settings improves communication skills of children and young people with PMLD.

**Methods:**

A multi-site pragmatic cluster randomised controlled trial comparing usual care with Intensive Interaction and usual care. Clusters will be educational settings. This study will recruit 330 participants (aged 3–25 years) with PMLD from 66 educational settings within Great Britain. Each participant will have a corresponding teacher, parent/carer, and interventionist. Potential participants will be screened by their educational setting for eligibility prior to giving informed consent. Data will be collected at baseline, 32 weeks, and 52 weeks post-randomisation and will assess health and educational outcomes including participants’ communication skills, behaviour, wellbeing, and quality of life. The primary outcome is communication skills, measured by the Communication Complexity Scale (CCS) at 32 weeks post-randomisation. Setting staff will video record an interaction with each participating child/young person. Communication will be coded by members of the research team blinded to allocation using the CCS.

**Discussion:**

This study addresses a much used but currently under-researched intervention and results will inform the support provided to children and young people with PMLD in their educational settings and at home.

**Trial registration:**

The trial was prospectively registered on the ISRCTN registry on 3rd May 2023 (registration number: ISRCTN81099965, https://www.isrctn.com/ISRCTN81099965).

## Introduction

### Background and rationale {9a}

Over 10,000 children and young people in England have profound and multiple learning disabilities (PMLD) [1]. All have multiple disabilities, the most significant being profound intellectual disability and great difficulty communicating [2]. They often have additional disabling conditions, e.g. physical disabilities limiting everyday tasks and restricting mobility; sensory impairments; processing difficulties; complex health needs. Their behaviours can often present as challenging [3].

Communication interventions aim to facilitate communication between people with PMLD and familiar partners [4]. There are various communication interventions in use but their clinical and cost-effectiveness are unclear. The James Lind Alliance Childhood Disability Research Priority Setting Partnership identified uncertainties about the timing and intensity of speech and language therapy as one of its top priorities [5]. A scoping study identified the need for speech and language therapies to be evaluated, with Intensive Interaction (II) the priority intervention for evaluation [6]. The Core and Essential Service Standards for Supporting People with PMLD state that staff should be trained in appropriate total communication approaches to maximise expressive and receptive communication for children and young people with PMLD [3].

Recent reviews have shown that II is widely used but currently lacks robust evidence for children and young people with PMLD in education settings [7–9]. This trial aims to establish the clinical and cost-effectiveness of II and close the evidence gap for this under-served population.

II works on early interaction abilities—how to enjoy being with other people—to relate, interact, know, understand and practise communication routines [10]. II teaches and develops communication skills, e.g. use and understanding of eye contact, facial expression, vocal mirroring and joint focus activities to build relationships [8]. The components of II are referred to as the Fundamentals of Communication and include learning to give brief attention to another person, sharing attention, developing shared attention in activities, taking turns and using and understanding eye contact and facial expressions [10]. Potential benefits are thus improved communication and quality of life. We will provide structured training in II to educational setting staff and parents/carers with coordinated activities developed jointly for each child/young person to be delivered within the educational setting and at home.

We do not anticipate that trial participants will be subject to any substantial risks during this study. There is a potential, however, due to the nature of PMLD, for the participants to experience some distress associated with the intervention. Educational staff will be advised to follow usual school safety policies so as to avoid any harm. Staff members involved will receive training in PMLD awareness as part of the II training.

The intervention may intrude on some children and young people’s existing routines, which might cause some distress. The design of this study ensures that the intervention delivery by setting interventionists will take place within the school day, i.e. as part of their existing scheduled activities, minimising this potential disruption. As this is a communication approach, the only additional burden on staff is the time commitment to undertake the training; however, we anticipate settings will welcome the opportunity for formal training and support.

In the absence of available evidence, we conducted a national survey of educational professionals and speech and language therapists (SaLTs) to establish current standard practice. The results show that standard practice includes a range of communication and interaction approaches, for example Talking Mats, Objects of Reference, Makaton Signing, Picture Exchange Communication System, Communication Passports/Profiles, II, visual support, music-based approaches, drama/storytelling approaches, Sensory Stories, Social Stories and switch-based approaches. Educational settings implement their approaches through a combination of individual sessions and embedding of the approach into daily interactions. Delivery of these approaches is mostly by teachers and teaching assistants, with SaLTs providing advice and support. Training received by staff is mixed, with training providers including SaLTs and Educational Psychology Services, organisations such as Mencap, other educational settings and in-house training; though for many of the approaches, no training is received.

The eligible population for this trial will be children/young people with PMLD and recruitment will be from geographic populations with high disease burden. We aim to recruit both specialist and mainstream educational settings with specialist facilities serving children/young people with PMLD between the ages of 3 and 25 years.

### Explanation for the choice of comparator {9b}

The trial investigates the effect of Intensive Interaction on the communication skills of children and young people with profound and multiple learning disabilities within their education setting. Both the intervention and control groups will continue to receive usual care and education from their educational setting. Usual care was selected as the comparator to reflect real-world, pragmatic conditions and to establish if structured II training provides a measurable benefit over the current highly variable standard practice. The intervention group will receive Intensive Interaction from people who have received specific training in II as an addition to usual care and education.

### Objectives {10}

#### Primary

The primary objective of the study is to establish whether Intensive Interaction delivered within educational settings improves communication skills of children and young people with PMLD compared to usual care at 32 weeks post-randomisation.

#### Secondary

The secondary objectives of the study are:

- To estimate the difference between groups with respect to a range of secondary outcomes over 52 weeks, specifically, children and young people’s educational outcomes, quality of life, social and emotional engagement, and behaviours that challenge; and parent/carer wellbeing.
- To estimate the cost-effectiveness of II relative to usual care alone.
- To examine acceptability and fidelity of the intervention to parents/carers of children and young people with PMLD and those delivering the intervention.

## Methods: patient and public involvement, and trial design

### Patient and public involvement {11}

Patient and Public Involvement and Engagement (PPIE) is integral to the strategic and operational delivery of this research. The PPIE group for this trial decided to be known as the Research Advisory Group (RAG), as they preferred not to refer to the children/young people with PMLD as patients. The RAG will comprise parent/carers of children and young people with PMLD; educational professionals from the Special Educational Needs sector and representatives from charitable organisations including Promoting a More Inclusive Society (PAMIS) and the Council for Disabled Children. Due to the significant needs of people with PMLD and the potential for significant distress, a person with PMLD will not directly be represented in the PPIE/RAG.

At all times, the PPIE lead will work with the PPIE/RAG members to ensure they are able to contribute within their capacity. Participation can be at many levels, i.e. reviewing documents by email, participating in meetings, participating by phone/video calls outside of meetings and joint participation in dissemination activities. Financial reimbursement is available in line with INVOLVE guidelines.

### Trial design {12}

INTERACT is a multi-site, two-arm, superiority, pragmatic cluster randomised controlled trial (cRCT) comparing Intensive Interaction delivered by specially trained educational professionals alongside usual care with a control group receiving usual care alone. For the purpose of this study, usual care is defined as the existing package of care and education routinely provided for a child or young person with PMLD within their educational setting. A statistician at YTU, who has no involvement with settings or in setting recruitment, will randomise educational settings to either the intervention or control arm, using a 1:1 allocation ratio. The trial includes an 18-month internal pilot, a process evaluation (including surveys, qualitative interviews, focus groups and an examination of intervention fidelity) and an economic evaluation. Educational settings will be randomly assigned to either the intervention group or usual care.

## Methods: participants, interventions and outcomes

### Trial setting {13}

The intervention will primarily be delivered in educational settings throughout Great Britain (England, Wales and Scotland) that are responsible for providing education to children and/or young people with PMLD. These will be a mixture of specialist schools and mainstream schools with specialist units or classes. We aim to recruit 66 educational settings. Parents/carers will also be offered training to deliver the intervention at home.

### Characteristics of the people who are needed for the trial

**Table:** 

Characteristic	The people we would expect to see included
Age	Children and young people aged between 3 and 25 years
Sex	Data will be collected from parent/carers regarding their children’s biological sex. Parent/carers will be asked to select from the following options: • Male • Female • Other
Gender	Given the population of children and young people with profound and multiple learning disabilities, only data regarding biological sex as described above will be collected
Race, ethnicity and ancestry	No restrictions will be placed on eligibility on the basis of race, ethnicity or ancestry. Parent/carers will be asked to describe their children’s ethnicity by selecting from the following categories: • White • Asian/Asian British • Black/Black British/Caribbean or African • Mixed or multiple ethnic groups • Other ethnic group
Socioeconomic status	We anticipate families from a range of socioeconomic backgrounds. Initially, we planned to capture data around parent/carer employment, education and training; however, after taking guidance from our PPIE group, these were removed to reduce the length of the questionnaire, and because they were viewed as unduly intrusive. We will collect data on whether or not parent/carer providing data are currently in employment, education or training, but nothing further
Geographic location	Recruitment will take place across Great Britain (England, Wales and Scotland). No restrictions will be in place with regard to geographical location. We anticipate a national spread of participants
Other characteristics relevant to the trial	No additional characteristics

### Eligibility criteria for participants {14a}

Educational settings must first agree to take part before any families of children/young people with PMLD can be recruited. Once a setting is confirmed eligible and wishing to take part, the recruitment of children/young people to participate will be based on the following criteria.

#### Inclusion criteria

- Age 3–25 years
- Attends an educational setting taking part in the trial.
- Have PMLD
- Parent/carer/person with legal responsibility willing to give consent/act as a consultee for child or young person
- Parent/carer willing to complete outcome measures
- Child or young person is expected to remain at the setting for the duration of the trial.

#### Exclusion criteria

- Have a degenerative condition
- Have dementia.

### Eligibility criteria for sites and those delivering interventions {14b}

The eligibility of educational settings will be assessed based on the following criteria.

#### Inclusion criteria

- Special Educational Needs and Disabilities (SEND) educational settings or mainstream educational settings with SEND units around Great Britain (England, Wales and Scotland)
- At least 5 children and young people meeting the eligibility criteria
- Capacity and willingness to agree to the requirements of participation outlined in a memorandum of understanding (MOU), which will ask settings to confirm that they have capacity and willingness to release staff to receive training; to deliver the intervention as intended with those children and young people participating in the trial; and to complete the outcome measures

#### Exclusion criteria

- Setting is unable to fulfil the requirements of the MOU
- Currently delivering weekly or more frequent II and staff have recently been formally trained in II within the last 12 months.
- Insufficient eligible children and young people.

Interventionists will be selected by the individual settings and are likely to be teaching assistants, teachers, or other staff employed at that setting. As this is a pragmatic community-based trial, the trial team has provided no restrictions as to who should or should not deliver the II, in terms of qualifications or other characteristics. Similarly, there are no specific requirements of parent/carers who choose to deliver II at home.

### Who will take informed consent? {32a}

Given the age and/or PMLD status of the target population, informed consent will not be obtained directly from the children and young people themselves. Appointed lead contacts at participating educational settings will be responsible for identifying eligible children and young people and distributing recruitment packs to parents/carers. Two processes will be in place to ensure consent depending on the age of the child/young person.

#### Children under the age of 16 years

For children under the age of 16, informed consent will be obtained from a parent/carer covering the child’s participation in the study, their willingness to complete questionnaires about the child and themselves, and whether they are willing to participate in the training and deliver II at home.

#### Young people aged 16 years and over

In the case of young people aged between 16 and 25 years, parents/carers will be asked to act as a consultee to confirm participation of the young person. They will also provide informed consent regarding their own participation in the study, including whether or not they agree to receive training and deliver II at home.

Although we will not seek consent or assent from the children and young people, interventionists will be advised not to proceed with intervention sessions if they appear to be distressing the child or young person.

In both cases, parents/carers will have the option of either completing a paper consent/consultee form or an e-consent/consultee form. Paper forms will be collected by appointed contacts within educational settings and securely transferred to the University of York for processing. E-consent will be obtained using a Qualtrics form. Additional consent may be required for looked-after children; for example, where the local authority has legal responsibility, in these circumstances, consent will also be sought from the child or young person’s appointed social worker.

#### Informed consent: process evaluation

The parent/carer consent/consultee form for trial participation will contain an optional statement about receiving information about taking part in an interview. Parents/carers who consent may be contacted and invited to interview. Informed consent will be obtained from all participants (e.g. parents/carers, educational practitioners, SaLTs) prior to the start of each interview using either e-consent or verbal consent methods depending on participant preference.

### Additional consent provisions for collection and use of participant data and biological specimens {32b}

No biological specimens will be collected, and no additional consents will be required besides those documented in {32a}.

## Intervention and comparator

### Intervention and comparator description {15a}

#### Intervention

Children and young people in the intervention arm of the trial will receive Intensive Interaction (II) in addition to their usual care. The II will be delivered by trained education practitioners in the child’s or young person’s setting and also by their parents/carers who may also choose to be trained. II is an intervention that aims to support people with PMLD to communicate more with their communication partners using any behaviours, e.g. eye gaze, vocalisations, body movements and changes in facial expressions. These behaviours may be hard to identify and not typically be viewed as communicative. II responds to these behaviours by understanding they are communicative and supporting the person to use them more as well as developing other behaviours that are interpreted as communicative. Central to II are the ‘Fundamentals of Communication’ [4] and these include always understanding the person with PMLD is an intentional communicator, II is enjoyable for all and the person with PMLD leads the II. There are ten II techniques the interventionist in the trial will learn (sharing personal space; making or exchanging eye contact/looking; exchanging facial expressions; behavioural mirroring; vocal echoing; turn taking; physical contact; joint focus activity; burst pause sequences and running commentary). There are 7 responses from the child or young person they learn to identify and respond to. These are encounter; awareness; attention and response; engagement; participation; involvement and participant-initiated activity. The communication partners use the ten techniques with the person with PMLD depending on the 7 responses the person shows. A key component is contingent responding where the communication partner follows the person’s lead in how the interaction develops [4]. This means the communication partner understands any behaviours of the person as communicative and uses the techniques to respond to these in order to shape and support more communication from the person. Communication partners engage in II with the person with PMLD as part of their everyday activities in that setting rather than scheduling a specific time of day. This is termed the ‘anytime, anywhere’ model in the II training. II is led by the person with PMLD and the communication partner learns to understand when the person starts to show they would like the II to come to an end. This means the length of II can be a few minutes or less or longer.

A cascade training model is used in the study. The II will be delivered by staff employed in the child/young person’s setting who have undertaken training developed by the research team in Intensive Interaction. These staff are usually teaching assistants, teachers, or Special Educational Needs Coordinators (SENCOs) who already work closely with the child and young person and are referred to hereafter as setting interventionists. SaLTs will be trained by the research team to support the setting interventionists throughout the training process, to consolidate their knowledge of Intensive Interaction and to deliver the Intensive Interaction to the children and young people. The SaLTs may already be located in the setting, e.g. employed directly by the setting or employed by the NHS to input into the setting. Where settings do not have a SaLT, they will receive support from a SaLT that has been assigned to support the setting by the research team. Parents/carers of the children and young people may also be trained to deliver II to their child/young people outside of the school setting, i.e. in their home environment. They are called parent/carer interventionists and are trained by the research team and supported by a SaLT associated with the child/young person’s setting or by a SaLT that has been assigned by the research team to support the setting. All SaLTs will receive regular supervision from members of the research team at the University of Sheffield.

The Intensive Interaction training to the SaLTs, setting and parent/carer interventionists will be delivered online to ensure accessibility for all. The content and delivery of training will be co-produced with the RAG.

Once the training is completed, the setting and parent/carer interventionists will deliver the II for a minimum of 18 weeks. The interventionists use an ‘anytime/anywhere’ model where the II is embedded into everyday activities where the duration is led by the child or young person. The II may occur for a few minutes several times a day or it could be once a day for 15 min. It is recommended that II occurs regularly and at least once a day. The setting and parent/carer interventions are asked to complete paper session record logs daily to identify the number and duration of the times the child/young person engaged in II with them and the II techniques the interventionist used and the responses they observed in the child/young person.

#### Comparator

Educational settings allocated to the control arm of the trial will be asked to continue to provide usual care and education to the participating children and young people at their setting. Settings in the control arm will receive training and support in II at the end of their involvement in the trial (i.e. after their 52-week follow-ups).

### Criteria for discontinuing or modifying allocated intervention/comparator {15b}

Given the population is children and young people lacking mental capacity, the decision to discontinue the intervention will be at the discretion of the educational professionals working directly with the children and young people within their educational setting. Educational professionals will be advised to discontinue the intervention if individual children/young people consistently show signs of distress during the intervention sessions. Professionals will be experienced in identifying signs of distress within children/young people with PMLD and with respect to the particular children and young people they are supporting in the study.

Parents/carers will also be able to request that the intervention is discontinued for their child at any time and without giving reasons.

As the comparator is usual care and education, there are no criteria for discontinuation.

### Strategies to improve adherence to intervention/comparator {15c}

Educational settings allocated to receive training and deliver II will receive support from a SaLT during their time in the trial. Training and resources will be provided online, and interventionists will be able to revisit these as required.

Interventionists, and parents/carers if they choose to deliver II at home, will be asked to complete a session record log each day during the intervention period to record key aspects of implementation. Whilst the trial team encourages the completion of session logs, it is anticipated that some settings or parent/carers may not action this request or may do so inconsistently. No attempts will be made to backfill any missing data as this would rely heavily on recall over a long period. Speech and language therapists appointed by the trial team to support the settings will meet with setting staff and parent/carers weekly for the first 4 weeks and then monthly thereafter to provide support and encourage adherence.

### Concomitant care permitted or prohibited during the trial {15d}

As II is complementary to the usual package of support and education received by children and young people with PMLD, no concomitant care will be prohibited during the course of the trial.

### Ancillary and post-trial care {34}

All participants will continue to receive their usual care and education following the trial. Settings that received the II training are able to continue delivering II at their discretion. Settings that are allocated to continue with care and education as usual will have the option to receive II training and support from a SaLT at the end of their trial period (52 weeks post-randomisation).

II is already widely used within educational settings and we do not anticipate any harm will come to trial participants as a consequence of receipt.

### Outcomes {16}

Data will be collected at baseline, 32- and 52–weeks post-randomisation from staff at educational settings and parents/carers.

#### Primary outcome

The primary outcome is the Communication Complexity Scale (CCS) [11–13] completed at 32 weeks post-randomisation. The CCS was developed to describe and measure the development of pre-intentional and intentional communication in individuals with developmental disabilities and minimal verbal skills. It has demonstrated good reliability and validity in children with intellectual and developmental disabilities [12]. The CCS consists of a 12-point scale and covers three levels of early communication (pre-intentional, intentional non-symbolic and intentional symbolic), with higher scores reflecting more complex communication. Lower scores describe no response (0), alerting (1) or pre-intentional communication acts (2–5), scores of 6–10 are for intentional non-symbolic communication acts and scores of 11–12 are for intentional symbolic communication acts (e.g. using speech, signs or symbols; 11).

In the trial, the CCS will be used to score naturalistic sessions [14]. At each time point, the associated teacher will be asked to video themselves and the participating child or young person interacting for approximately 10 min following guidance provided by the trial team. Video recordings will be scored using the CCS by members of the research team who are blind to allocation. In line with training and guidance from the CCS developers, each video will be split into 30-s intervals, and each interval will be allocated a score of 0–12, i.e. the score for the highest scoring communication act within that interval. For scores of 6–12 (i.e. intentional communication), a function score will be allocated according to whether the function of the communication act was for ‘behaviour regulation’ (i.e. to bring about a specific result by requesting or protesting) or ‘joint attention’ (i.e. to direct a communication partner’s attention for social commenting; 11). An ‘optimal score’ will be calculated at each time point by averaging the three highest CCS scores [11]. The optimal score at 32 weeks post-randomisation will be the primary outcome and the optimal score at 52 weeks post-randomisation will be a secondary outcome.

#### Secondary outcomes

Secondary outcomes will also be collected at baseline, 32- and 52- weeks post-randomisation. These are outlined below, grouped by respondent.

Parent questionnaires:

- Quality of Life–Profound Multiple Disabilities (QOL-PMD): measuring quality of life [15]: mean difference in total scores between groups at 32 and 52 weeks adjusting for baseline
- Short Warwick-Edinburgh Mental Well-being Scale (SWEMWBS): measuring parental wellbeing [16]: mean difference in total scores between groups at 32 and 52 weeks adjusting for baseline
- Mood, Interest and Pleasure Questionnaire—Short Form (MIPQ-S) [17]: mean difference in total scores between groups at 32 and 52 weeks adjusting for baseline

Teacher questionnaires:

- Modified Routes for Learning [18]: mean difference in total scores between groups at 32 and 52 weeks adjusting for baseline
- Education Health and Care Plan (EHCP) Changes: estimated treatment effects at 32- and 52–weeks and overall will be presented as an odds ratio
- Behaviour Problems Inventory-Short Form (BPI-S) [19]: mean difference in total scores between groups at 32 and 52 weeks adjusting for baseline

#### Health economics outcomes

The following outcome measures will be collected for the purpose of the economic evaluation. How these will be analysed is detailed under item 27a.

Parent questionnaires:

- Child Health Utility-9D (CHU-9D) proxy version: measuring quality of life [20]
- EQ-5D-Y proxy version: measuring quality of life [21].
- The Carer Experience Scale: measuring parental quality of life [21]
- Bespoke Resource Use Questionnaire: measuring personal, healthcare, social, educational and societal resource use [20]

Teacher questionnaires:

- EQ-5D-Y proxy version: measuring quality of life [21]
- Bespoke Resource Use Questionnaire [22]: measuring resource use within the educational setting

#### Other collected variables

At baseline, teachers will be asked whether each child/young person has a sight impairment, hearing impairment, epilepsy diagnosis, whether epilepsy is controlled, whether the child or young person has a diagnosis of autism, and whether the child/young person receives their nutrition orally. At baseline, parents/carers will be asked to report the child/young person’s biological sex, ethnicity and living arrangements, as well as their own gender, ethnicity and relationship status.

### Harms {17}

Given the nature of the intervention being evaluated, no adverse events (AE) or serious adverse events (SAE) that are unexpected and related are anticipated. However, as the intervention sessions will be novel for the children and young people, and they may intrude on some children’s existing routines, this might cause distress for some children/young people with PMLD.

We will take a non-systematic approach to adverse event approaching, rather we will ask SaLTs, interventionists based at the educational settings and parents/carers in both arms of the trial to report if they have a concern about a participating child or young person or they experience an incident, event, negative change (e.g. to their communication or behaviour) or out of character distress that may be related to taking part in the INTERACT trial.

Following consultation with our research advisory group and drawing on the trial team’s clinical knowledge, we will collect data regarding specific adverse events as follows:

- New or worsening behaviour that challenges
- Exhibited a level of distress that is out of character
- Reduced levels of communication.
- Injured at home or at setting.
- Hospitalised unexpectedly for any reason or had a planned hospital stay unexpectedly lengthened.
- Negative changes to sleep patterns
- Reduced access to respite due to changes in behaviour.
- Moved to a different educational setting unexpectedly due to additional needs.
- New involvement from Child and Adolescent Mental Health Services.
- Other concerns (please specify)

Participating SaLTs, interventionists and parent/carers will be provided with the list above and asked to report any occurrences by telephone to York Trials Unit using a dedicated trial telephone number. The trial team will then complete an adverse event form and follow up as appropriate.

### Participant timeline {18}

Participants will be enrolled in the study and the baseline assessment completed in the autumn term (September to December). The intervention will be delivered during spring and summer terms (January to May). Data will be collected 32 weeks post-randomisation (June–July) and 52 weeks post-randomisation (November–December).

### Sample size {19}

The primary outcome is the CCS at 32 weeks. A study utilising the CCS for communication skills of children with autism spectrum disorder who were minimally verbal [13] indicated that a 1-point increase in scores is a minimal clinically important difference (MCID). Assuming a MCID of one point on the CCS [13], a standard deviation of 2.08 [12], 90% power, 5% alpha, 20% loss to follow-up at the child/young person level, 10% drop out at the educational setting level, ICC = 0.09 (calculated from a similar trial published by the trial team, [22] and an average cluster size of 5, we would need to randomise 66 educational settings (330 children and young people). Sample size calculations were conducted in Stata v17 using the command clustersampsi. The Stata code used to determine the number of educational settings is given as follows:

- clustersampsi, mu1(0) mu2(1) sd1(2.08) sd2(2.08) m(4) rho(0.09) beta(0.9)

with further adjustment to the number of educational settings to allow for up to 10% attrition.

### Recruitment {20}

The study aims to recruit 66 educational settings and 330 children/young people with PMLD from Great Britain. Recruitment will take place in three waves during 2023, 2024 and 2025. Data will be collected from a nominated teacher and a nominated parent/carer for each participating child/young person. For those allocated to receive the intervention, data will also be collected from the child/young person’s main communication partner (referred to as the interventionist). Recruitment will be both targeted and opportunistic.

Educational settings serving children and young people with PMLD across Great Britain will be contacted by email with a recruitment flyer containing basic information about the trial and a link to the trial website.

We will also publicise the study on social media platforms, such as X, Facebook and LinkedIn. The Council for Disabled Children and PAMIS may also promote the materials through their connections, webpages and newsletters.

Interested parties will be required to complete an online expression of interest form which acts as a screening tool to ensure settings meet the required inclusion criteria. Those that express an interest in participating and are confirmed eligible will be sent full details about the study, more specific information about the population characteristics, as well as a memorandum of understanding (MOU) and Data Sharing Agreement (DSA) to complete.

Following the receipt of a valid MOU and DSA, settings will be provided with recruitment packs to distribute to families. Families will be given the option of completing an online consent form or a paper copy. Paper copies will be securely sent from the educational setting to York Trials Unit for processing.

#### Recruitment to the process evaluation

As part of the process evaluation, interviews will be conducted with key stakeholders responsible for delivering the intervention, including SaLTs, educational practitioners and the parents/carers of participating children. The research team will work closely with staff in educational settings to ensure that a diverse sample of parents/carers and staff involved in delivering the intervention is selected according to the following criteria: staff group (education practitioners, SaLTs), educational setting size and type (SEND, mainstream educational settings with SEND units), geographical location, PMLD, and child’s age, gender and ethnicity. The preparatory work undertaken to identify ‘target educational settings’ will be crucial to our sampling by illustrating the sites where we may wish to focus our efforts to achieve this diversity. SaLTs, educational practitioners and parents/carers will be invited by the qualitative research team via email/phone call to take part in a qualitative interview including a copy of the Participant Information Sheet and will be sent an online consent form if they wish to participate.

## Assignment of interventions: randomisation

### Sequence generation: who will generate the sequence {21a}

A statistician at York Trials Unit, who has no involvement with settings or in setting recruitment, will randomise educational settings to either receive II alongside usual care or usual care alone. Once a setting is considered ready to randomise, i.e. baseline data collection is complete, the trial manager or trial coordinator will enter relevant details about that setting for randomisation onto a spreadsheet prepared by the trial statistician. The statistician will then randomise the setting as described in section {21b}.

### Sequence generation: type of randomisation {21b}

Educational settings will be randomised to either receive II alongside usual care or usual care alone, using a 1:1 allocation ratio. Educational settings will be randomised via minimisation to ensure the best possible balance across educational setting characteristics within each group. A dedicated computer programme, MinimPY [23], will be used with the following minimisation factors:

- Age range of educational setting (primary age only (up to 11 years); non-primary (age 11 +); all age educational settings)
- Residential status of educational settings (residential; non-residential)
- Number of children and young people with PMLD (latest available data dichotomised at the median for recruited educational setting in the first batch to be randomised)
- Local authority region (East Midlands, East of England, London, North East, North West, Scotland or Wales, South East, South West, West Midlands, Yorkshire and Humber)

### Allocation concealment mechanism {22}

Randomisation will take place after eligibility has been confirmed, consent has been obtained, and baseline data collection from an educational professional has been completed. Randomisation and allocation to treatment groups will be conducted by the trial statistician who will have had no prior involvement in the recruitment or set-up of settings.

### Implementation {23}

The allocation sequence will be generated by the trial statistician who is independent of the trial management team and has no contact with participants or educational settings. The trial statistician will notify the trial management team of the randomisation outcome. The trial management team will notify educational settings and parent/carers. Settings allocated to receive II training will receive further communication from the trial team to confirm whether an in-house SaLT is available to receive training or if an external SaLT is required. Trained SaLTs will be responsible for cascading training to school staff and providing support.

## Assignment of interventions: blinding

### Who will be blinded {24a}

Due to the nature of the intervention, it will not be possible to blind parents/carers or staff within educational settings to allocation. Members of the research team responsible for coding the CCS data will be blinded to allocation. Any instances of unblinding will be recorded using a bespoke form. In such instances, another member of the research team will be responsible for coding wherever possible. The Data Monitoring Committee (DMC) will have access to the unblinded data during the trial.

### How will be blinding be achieved {24b}

Participants will be identified using a unique participant ID. Blinded members of the team will not have access to locations where unblinded data is saved. Educational settings will be reminded not to unblind the blinded team members.

Should a team member become unblinded to a settings’ allocation, an alternative team member will be allocated to code the data for that setting.

### Procedure for unblinding if needed {24c}

As only research team members responsible for coding the CCS data will be blinded, there will be no requirement for unblinding.

## Data collection and management

### Plans for assessment and collection of outcomes {25a}

**Fig. 1:**
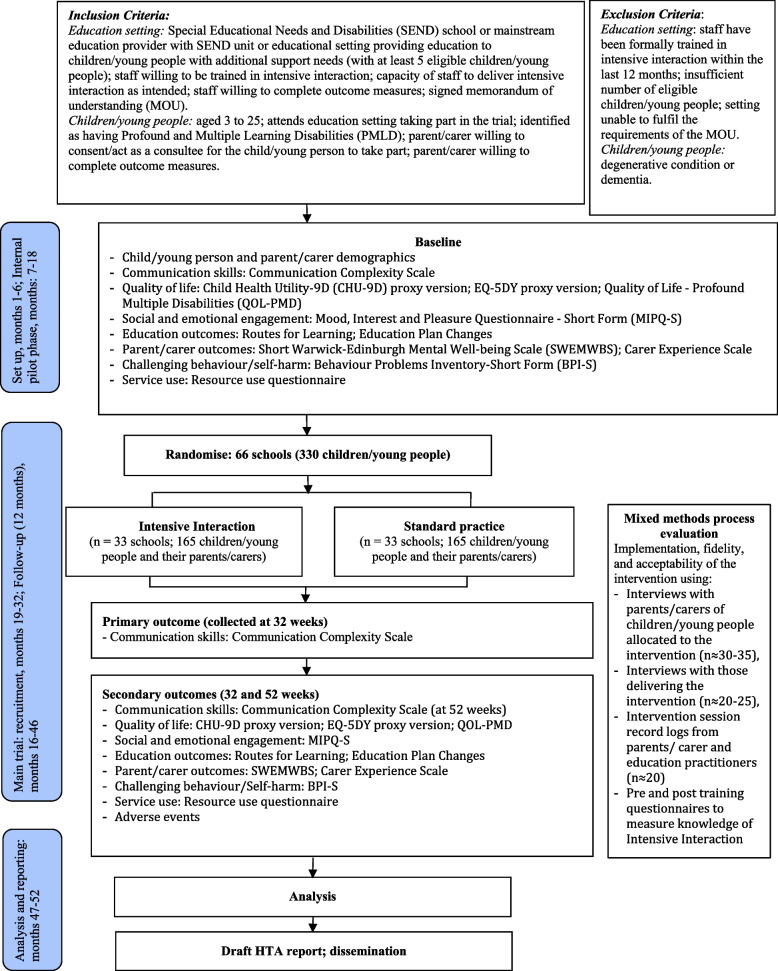
Participant flow through the study

**Fig. 2:**
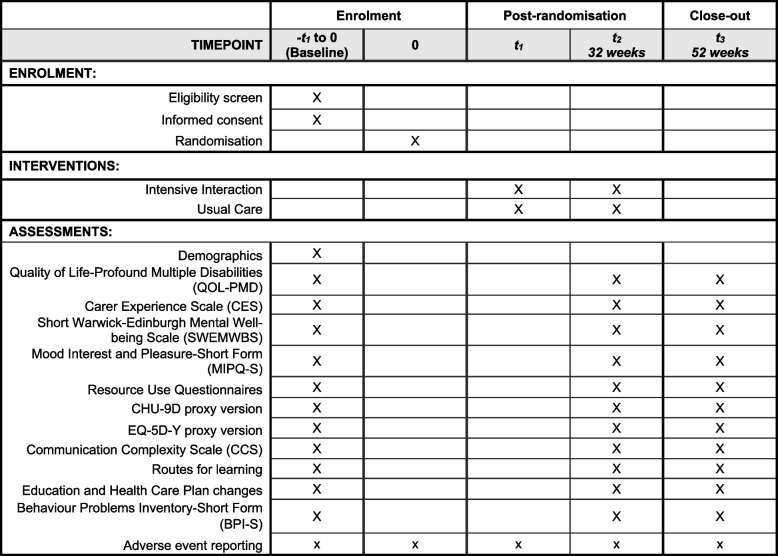
Participant timeline: schedule of enrolment, interventions, and assessments

Data will be collected at baseline, 32- and 52-weeks post-randomisation from both teachers and parents/carers. The full trial timeline with corresponding activities is presented in Fig. 1. Participant specific activities are presented in a SPIRIT table in Fig. 2 [24]. At baseline, parents/carers will complete a questionnaire online or over the phone with a research assistant. The questionnaire includes some demographic questions about the child/young person and the parent/carer as well as the following outcome measures: QoL-PMD [15], SWEMWBS [16], CHU-9D [20], EQ-5D-Y proxy [21], CES [25], bespoke resource use adapted from a previous study [26].

Similarly, teachers will also complete an online baseline questionnaire, again asking some demographic questions as well as the following outcome measures: Routes for Learning [18], BPI-S [19]. The teacher baseline questionnaire will also ask questions around education plan changes and in-school resource use.

In addition to the questionnaire, teachers will also be asked to record a video of themselves interacting with the child/young person. These videos will be scored using the CCS by trained coders [12, 13]. Three members of the trial team received training directly from the instrument development team at the University of Kansas. This comprised some independent learning using materials provided by the Kansas team as well as in-person training via videoconferencing. This training was then cascaded to an additional member of the team. Inter-rater reliability will be monitored throughout the coding process with at least 10% of videos being coded by two members of the team. Disagreements will be resolved by way of a discussion.

Parent/carers and teachers will complete questionnaires comprising the same outcome measures at 32- and 52- weeks post-randomisation. Teachers will also complete videos at these time points to be scored using the CCS.

Interventionists in school and at home will be given a session record log to record their use of II. This will be returned to the research team at the end of the intervention period and will be used to understand fidelity.

### Plans to promote participant retention and complete follow-up {25b}

To enhance participant retention and encourage the completion of follow-ups, newsletters will be circulated at appropriate intervals and shortly ahead of follow-up windows. Parents/carers will be offered online or over the phone follow-ups to ensure access. Thank-you payments of £15 will be offered to parents/carers upon completion of the final follow-up questionnaire. Similarly, educational settings will be offered thank-you payments of £350. Educational settings allocated to the control group will be eligible to receive the II training and support after the final follow-ups have been completed, which may act as an additional incentive.

Withdrawal can occur at any point during the study at the request of the parents/carers or educational settings. To try and improve some level of retention, withdrawals will be categorised into one of five categories:

1. Withdrawal from the intervention (individual level)
  A parent/carer or setting may choose to withdraw a child or young person from receiving the intervention at any time. Data will continue to be collected with continued agreement from the parent/carer and setting.
2. Withdrawal from the intervention (setting level)
  A setting may choose to withdraw from delivering the intervention at any time. Data will continue to be collected with continued agreement from the parent/carer and setting.
3. Withdrawal from parent/carer data collection (individual level)
  A parent/carer may choose to withdraw from providing data at any time. Intervention delivery and data collection from the child’s or young person’s educational setting will continue with agreement from the parent/carer and setting.
4. Full withdrawal (individual level)
  A parent/carer or setting may choose to fully withdraw a child or young person from receiving the intervention and from data collection at any time. Data will be retained in the dataset with continued agreement from the parent and educational setting.
5. Full withdrawal (setting level)
  A setting may choose to fully withdraw from participating in the trial at any time. No further data will be collected from the setting. Parent/carer data collection will also be stopped as the team rely on the setting for information communication; for example, if a child was to sadly pass away, the team is usually notified by the setting so that parent/carer communication can be stopped. Data will be retained in the dataset with continued agreement from the parent and educational setting.

### Data management {26}

Data collected as part of this trial includes questionnaires, surveys, qualitative data from interviews and video data (audio and visual). REDCap and Qualtrics will be used for electronic data capture. Validation will be built into these systems to ensure accuracy, completeness and reliability. Interviews and focus groups will be transcribed verbatim by a service compliant with UK GDPR. Data integrity is maintained through regular validation reports with all discrepancies fully documented and resolved.

The Universities of York, Kent and Sheffield will be involved in the handling of data. All data will be restricted to those individuals who require access in order to fulfil the responsibilities of their role in the trial.

All data will be kept strictly confidential with access restricted to necessary team members only. Video data (audio and visual) will be stored at the University of Kent or the University of York. All questionnaire data will be collected electronically using a mixture of REDCap and Qualtrics either via self-completion or with assistance from a trial support officer over the phone. All electronic questionnaire data will be stored on a secure password-protected server located at the University of York. Any data captured on paper, e.g. session record logs, will be entered onto the electronic system, after which paper copies will be stored in a locked filing cabinet in a room with restricted access at the University of York. Paper copies of questionnaires will be destroyed at the end of the trial (expected June 2027). All personal data will be destroyed 10 years after completion of the trial whilst anonymised data will be retained indefinitely.

### Confidentiality {33}

All information collected during the course of the study will be kept strictly confidential. All identifiable participant data will be coded, pseudonymised by participant number in all manual and electronic files and no participant identifiable data will be transferred from the database to the statistician. Output for analysis will be generated in a format, and at intervals, to be agreed between YTU and the chief investigators. Data will be stored on University of York computers with restricted access; these will all be password-protected.

All data will be collected and retained in accordance with the UK General Data Protection Regulation, Data Protection Act 2018 and YTU Standard Operating Procedures (SOPs).

## Statistical methods

### Statistical methods for primary and secondary outcomes {27a}

Statistical analyses will be conducted using Stata v19 or later (to be confirmed in the final report) and will be reported in line with the Consolidated Standards of Reporting Trials (CONSORT) 2025 statement, adjusted for a cluster RCT. The analysis will be undertaken according to the principles of intention to treat (ITT). The flow of settings and children/young people through the trial will be presented in a CONSORT diagram. The number of settings approached, recruited and randomised will be presented alongside the number of children/young people and parents/carers identified as eligible for the evaluation, approached and recruited. The number of educational settings and/or participants who withdraw from the intervention and/or trial will be presented, alongside reasons for withdrawal, if available. All baseline data will be summarised descriptively by group. No formal statistical comparisons for baseline imbalance will be undertaken. Continuous data will be presented using a mean and standard deviation and categorical data will be presented using a count and a percentage. All outcomes will be reported descriptively at all follow-up time points.

#### Primary analysis

The primary analysis will compare the optimal CCS score between groups using a covariance pattern mixed linear model, incorporating all post-randomisation time points (32 and 52 weeks). The model will adjust for minimisation factors, baseline optimal CCS score, time, treatment arm, treatment-by-time interaction and other relevant baseline covariates as fixed effects, with educational setting and child/young person as random effects to account for the clustering by setting and repeated observations per participant. The correlation of observations within participants over time will be modelled by a covariance structure. The Akaike information criterion will be used to compare models specifying different covariance structures (smaller values preferred). Model assumptions will be checked, and if they are in doubt, the data will be transformed prior to analysis. The treatment effect overall and at both time points (32- and 52 weeks) will be extracted in the form of an adjusted mean difference, 95% confidence interval and *p* value. The primary timepoint of interest is 32 weeks post-randomisation.

#### Secondary analyses

A similar approach to the primary analysis will be adopted for the secondary outcomes, adjusting for baseline scores where appropriate.

#### Adverse events

Adverse events will be summarised descriptively. The frequency of each of the pre-specified adverse events will be reported by trial arm.

#### Health economic analysis

The cost-effectiveness of II versus care as usual for children and young people with PMLD will be evaluated over a 12-month period using a within-trial cost-utility analysis, which will assess a wide range of costs and health outcomes of II and report the results separately, so decision makers can form their own opinion on the most relevant results for their local context. The costing perspective of the NHS and Personal Social Services (NHS/PSS) and Education Services will be taken in the base case, with a set of scenario analyses conducted from both the NHS/PSS and the societal perspectives. Utilities collected via EQ-5D-Y (proxy version) will be used to generate quality-adjusted life years (QALYs), which will be taken in the base case. Other effectiveness outcomes that will be assessed include the QALYs measured by the CHU-9D (proxy version), CES and CCS (primary trial outcome). The cost-effectiveness results will be presented in terms of the incremental cost-effectiveness ratio (ICER) for II compared with care as usual. Health outcomes, resource use and cost data will be collected using parent/carer self-completed questionnaires (at baseline, 32 weeks and 52 weeks), and using the information recorded during the trial by teachers and interventionists.

The cost of delivering the II intervention will be estimated using a bottom-up approach, thereby incorporating the time spent by professionals training (for both trainers and trainees) and delivering the intervention (to plan and undertake II), as well as the costs of all associated resources and materials. Unit costs, obtained from established costing sources (e.g. NHS Reference costs, PSSRU), will be applied to each resource item to generate total cost estimates for each participating child/young person. Further costs feeding into the secondary analysis, such as societal perspective, will be collected to explore the impact of private expenditures (i.e. out-of-pocket medication expenditure, travel costs for appointments) and parent/carer time off work (for caring, attending appointments).

Mean within-trial estimates of costs and utilities will be generated using regression methods, adjusting for key covariates (including baseline utility), and allowing for any correlation between costs and utilities. Missing data patterns will be analysed to guide the multiple imputation methods used to deal with missing data [27]. Analyses will take an intention-to-treat approach, with findings presented in terms of mean costs and effects for both groups, as incremental cost-effectiveness ratios at 12 months. A non-parametric bootstrap re-sampling method will be used to produce confidence intervals around the cost and QALY differences and the ICER. The bootstrapped results will be presented in cost-effectiveness planes and cost-effectiveness acceptability curves [28]. A set of sensitivity analyses will be conducted to explore the robustness of the cost-effectiveness results. The NICE (2013) Guide to the Methods of Technology Appraisal will be followed wherever possible [29], and a pre-specified health economics analysis plan will be agreed with the trial’s independent groups and signed off by the chief investigators.

### Who will be included in each analysis {27b}

The analysis will be undertaken according to the principles of intention to treat (ITT). All randomised participants, with the exception of those that have fully withdrawn and have asked for their data to be removed from the dataset, will be included in the analysis.

### How missing data will be handled in the analysis {27c}

We will assess the impact of missing data on the study results. Case and item missing data will be examined, and multiple imputation methods may be used to reduce bias due to any missing responses in the analyses. Where appropriate, modelling methods that generate robust standard errors in the presence of missing data will be considered.

### Methods for additional analyses (e.g. subgroup analyses) {27d}

A subgroup analysis will consider whether there is evidence of a differential intervention effect depending on the age of the child and/or young person (primary vs non-primary age provision). This will be assessed by repeating the primary analysis but including an indicator variable for whether the child and/or young person is attending a primary educational setting or a non-primary educational setting at baseline as a covariate plus an interaction term between treatment allocation and age provision.

#### Process evaluation analyses

Qualitative data will be analysed using thematic analysis, which will involve flexibly and iteratively engaging in detailed familiarisation, generating initial codes, searching for themes, reviewing themes, defining and naming themes and data reporting. Throughout the analysis, theme development will be largely deductive and informed but not limited to the broad categories as described in the Hasson fidelity model (context, coverage/recruitment/evaluation of adherence, participant responsiveness, intervention complexity and strategies to facilitate implementation) [30]. Training questionnaires will be analysed descriptively and summary statistics presented. Data collected through intervention delivery session record logs will be analysed using content analysis [31].

Quantitative and qualitative data will be analysed separately using the methods described above. Data will then be integrated using Hasson’s framework to give an overall understanding of intervention fidelity and acceptability [30, 32]. During this final mixed methods analysis, we will seek to identify key areas for consideration and/or adaptation to inform any future implementation of II should the intervention be proven to be effective.

### Interim analyses {28b}

For the first 18 months, we will conduct an internal pilot with 22 educational settings, each with approximately 5 pupils participating, yielding a total of around 110 pupils. This would account for around a third of the overall proposed sample. The internal pilot will be reviewed by the trial oversight committees and the funder to determine whether the study progresses to the full trial. We propose a traffic-light system for progression criteria as per Table 1.

**Table 1:**
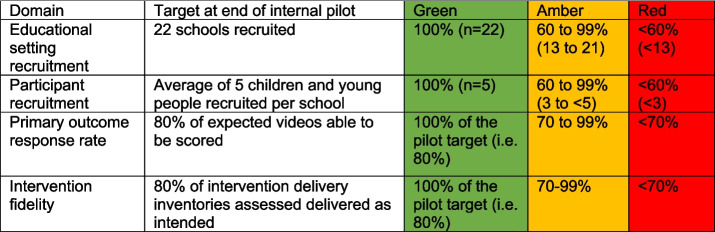
Criteria for progress to full trial

### Protocol and statistical analysis plan {5}

A statistical analysis plan (SAP) and a health economics analysis plan (HEAP) will be developed ahead of the analysis. These will be reviewed by the trial oversight committees, the Trial Steering Committee and the Data Monitoring Committee. Copies of the SAP and HEAP will be available from the trial team upon request and will also be submitted as an update to the protocol.

## Oversight and monitoring

### Composition of the coordinating centre and trial steering committee {3d}

York Trials Unit, University of York, is the coordinating centre for INTERACT. The team comprises a trial manager, trial coordinators, trial support officers, statisticians and data managers who work alongside the chief investigators and the sponsor. The coordinating team will be responsible for ensuring all relevant approvals are in place, recruiting settings and supporting data collection processes. The data management team will process and check data against the validation criteria agreed. In addition to the coordinating centre, a team at the University of Sheffield will be responsible for leading the training of the speech and language therapists and supporting the training of educational settings. A team at the University of Kent will be responsible for obtaining video data and scoring this against the Communication Complexity Scale (CCS), which is the primary outcome for the trial.

The Trial Management Group (TMG) comprising the coordinating team, implementation team at Sheffield, wider grant co-applicants including representatives from charity-based partners, statisticians, health economists, qualitative researchers and the chief investigators will meet monthly to review trial progress.

The Trial Steering Committee (TSC) comprises expert researchers in communication, an independent statistician, a health economist, and a qualitative researcher, as well as public members. The TSC will review trial progress every 6 months throughout.

A Research Advisory Group (RAG) will meet regularly to input on trial processes and progress.

### Composition of the data monitoring committee, its role and reporting structure {28a}

The Data Monitoring Committee (DMC) comprises an expert researcher in communication, an independent statistician, and an independent health economist. The DMC will review trial progress every 6 months throughout the trial. To improve efficiency, it has been agreed with the Chairs of the DMC and TSC that oversight meetings will be conducted jointly with members of both committees attending the same meeting. In the event that meetings have to take place separately due to logistics, the DMC will make recommendations regarding safety or any other issues in writing to the TSC.

### Frequency and plans for auditing trial conduct {29}

No on-site monitoring will be conducted in this trial due to the low-risk behavioural nature of the intervention being evaluated and the setting being community-based educational settings. Ongoing in-house quality checks and continuous monitoring by the coordinating centre will be applied.

### Protocol amendments {31}

Any amendments will be made following REC procedures as per standard practice. Educational settings and other relevant parties will be informed of any relevant changes via email and will receive copies of any associated documentation. In the event that parents/carers of participating children and young people need to be notified of an amendment, a letter detailing these changes would be prepared and sent via email (as the team are not collecting postal addresses). This letter would be sent alongside the amendment to the REC for approval prior to circulation. Where the team does not hold a valid email address, educational settings will be asked to pass the letter to parents/carers using their usual setting-to-parent communication pathways (e.g. physical letters, setting mobile phone app).

The public registry, ISRCTN, will be updated following any amendments.

### Dissemination policy {8}

Our dissemination plans involve working closely with the full range of stakeholders for whom the study will be of relevance and interest. This will include clinical and educational professionals working with children and young people with PMLD as well as parents/carers and voluntary sector organisations. Activities will include engagement throughout the study, ultimately informing a range of outputs to ensure the learning from this study effects the appropriate change for the benefit of those with, and those caring for children and young people with PMLD.

In addition to producing the HTA report, we will publish open access articles in relevant academic journals covering the disciplines of SaLT and psychology. We will also prepare articles for practitioner journals and magazines and present to conferences such as the National Association for Special Educational Needs (NASEN) which supports all education practitioners, International Association for the Scientific Study of Intellectual and Developmental Disabilities, European Academy of Childhood Disability.

A plain English summary bulletin will be sent to all participating educational settings and the parents/carers of the children and young people taking part. This will be translated into other languages as required. The bulletin will also be shared with other non-participating special education educational settings, mainstream educational settings that cater for PMLD pupils and other stakeholders, including the applicants’ extensive contacts with professional organisations such as the Royal College of Speech and Language Therapists, their Clinical Excellence Networks and other policy making bodies. Awareness raising work with educational settings and local authorities will aim to underpin and support subsequent implementation of any revised guidance.

We will aim to distribute study newsletters to participating settings that can also be shared with parents/carers at appropriate intervals.

## Discussion

To our knowledge, this is the first large pragmatic cluster RCT to be undertaken examining the effectiveness and cost-effectiveness of II in educational settings for children and young people with PMLD.

Delivering research within educational settings presents numerous challenges; for example, unlike in NHS settings, schools do not have dedicated capacity to facilitate research. Teacher’s time is already in high demand, and fitting in additional research activities such as questionnaire completion is difficult. Additionally, recruitment must be school-led, and parent/carer details cannot be shared with the research team until they have given consent. This means that the coordinating research team is removed from the initial screening and recruitment process until the parent/carers have either agreed to take part or until they contact the research team directly. In school-based research, staff and child absences can cause difficulties with regard to implementation of interventions and data collection, particularly within this population where children are often unwell.

Another potential challenge is parent/carer implementation of II at home. Whilst our preference is to include parents/carers in the training process so that II can be used at home, this poses an additional challenge as parents/carers may lack capacity to fit the training programme around their other responsibilities. As such, we have designed the trial so that parents/carers can opt-in to training during the trial and will have access to training materials after the trial to engage with at their convenience. As described above, we will measure parent/carer engagement with training and implementation of II at home during the course of the trial.

Although II is already recommended by SaLTs as a way of improving communications, the lack of reliable evidence of effectiveness for II and variation in delivery may be hampering wide and consistent uptake in practice. Our planned engagement and dissemination activities aim to raise awareness of this study with all relevant stakeholder groups, with the ultimate goal of ensuring a change in practice nationally. We will, post-study, continue to use our expertise in dissemination and, depending on the outcome of the study, ensure that policy makers are informed as needed. Clinical colleagues and our network of clinical and parent advocates will work on ensuring implementation is facilitated nationally. The anticipated change in practice should ensure a positive impact for teachers, parents/carers, and children/young people with PMLD as improved communication is known to improve quality of life.

## Trial status

The current version of the protocol is version 1.2 01/05/2025. Recruitment for settings opened on the 24th May 2023 with the first participants randomised on the 11th December 2023. The trial is currently still open for recruitment, with recruitment due to end on the 19th December 2025. Final follow-ups for the last participants randomised will be completed in December 2026, with results being available late 2027.

## Data Availability

The datasets generated and/or analysed during the current study will be available upon reasonable request from York Trials Unit following the completion of the trial and publication of trial results. Requests will be considered by the Trial Management Group on a case-by-case basis and approval given by the Co-Chief Investigators. Data will be made available for secondary analyses, and only anonymised data will be provided.

## Abbreviations

AE: Adverse event
BPI-S: Behaviour Problems Inventory-Short Form
CCS: Communication Complexity Scale
CES: Carer Experience Scale
CHU-9D: Child Health Utility-9D
CI: Chief investigator
CONSORT: Consolidated Standards Of Reporting Trials
DMC: Data Monitoring Committee
DSA: Data Sharing Agreement
EHCP: Education Health Care Plan
EQ-5D-Y: European Quality of Life-5 Dimension-Youth
GP: General practitioner
HEAP: Health economics analysis plan
HTA: Health Technology Assessment
ICER: Incremental cost-effectiveness ratio
INCLUDE: INCLUDE is an initiative from the UK’s National Institute for Health Research (NIHR) that aims to improve trial delivery for under-served groups
Interventionist: A member of setting staff or parent/carer who will deliver the intervention
II: Intensive Interaction
ITT: Intention to treat
MOU: Memorandum of understanding
NASEN: National Association for Special Educational Needs
NHS: National Health Service
NICE: National Institute for Health and Care Excellence
NIHR: National Institute for Health and Care Research
PAMIS: Promoting A More Inclusive Society
PIS: Participant Information Sheet
PMLD: Profound and multiple learning disabilities
PPIE: Patient and Public Involvement and Engagement
PSS: Personal Social Services
QALY: Quality-adjusted life years
QOL-PMD: Quality of Life-Profound Multiple Disabilities Questionnaire
RAG: Research Advisory Group
RCT: Randomised controlled trial
SAE: Serious adverse events
SaLT(s): Speech and language therapist(s)
SAP: Statistical analysis plan
SENCO(s): Special Educational Needs Co-ordinator(s)
SEND: Special Educational Needs and Disabilities
SOP: Standard Operating Procedure
SWEMWBS: Short Warwick-Edinburgh Mental Well-being Scale
TA: Teaching assistant
TMG: Trial Management Group
TSC: Trial Steering Committee
UK: United Kingdom
YTU: York Trials Unit
